# Synergistic Effect of *Passiflora incarnata* L., Herba and Cognitive Behavioural Therapy in the Management of Benzodiazepine Misuse

**DOI:** 10.3390/ph19010141

**Published:** 2026-01-14

**Authors:** Matteo Carminati, Mattia Tondello, Martina Zappia, Raffaella Zanardi

**Affiliations:** 1 Mood Disorder Unit, IRCCS San Raffaele Hospital, 20132 Milan, Italy; 2 Department of Clinical Neurosciences, Vita-Salute San Raffaele University, 20132 Milan, Italy

**Keywords:** benzodiazepine addiction, benzodiazepine tapering, *Passiflora incarnata*, cognitive-behavioural therapy, GABAergic modulation, depressive disorders, anxiety disorders, dependence

## Abstract

**Background/Objectives**. Chronic benzodiazepine (BDZ) use is frequently maintained beyond recommended durations due to neuroadaptation, psychological dependence, and withdrawal-related issues. *Passiflora incarnata* L., herba (*P. incarnata*) has shown anxiolytic and GABAergic activity that may mitigate withdrawal symptoms, while cognitive-behavioural therapy (CBT) targets maladaptive beliefs and behaviours sustaining BDZ misuse. This study investigates the independent and interactive effects of *P. incarnata* and CBT on BDZ dose reduction during a three-month tapering program. **Methods**. This retrospective observational study included 186 outpatients with anxiety or depressive disorders in clinical remission undergoing BDZ tapering, of whom 93 received a dry extract of *P. incarnata* as adjunctive treatment and 93, matched for diagnosis, age and sex, followed a standard tapering protocol. BDZ doses were assessed at baseline and three months. CBT was recorded as a binary variable based on the information documented in the medical records. An ANCOVA was performed to assess the impact of CBT and *P. incarnata* on BDZ reduction (change in mg diazepam equivalents), adjusting for sex, age, education, baseline anxiety and depression scores, initial BDZ and antidepressant dosage. A subgroup analysis was conducted to investigate the role of *P. incarnata* dosage in BDZ reduction. **Results**. Both CBT and *P. incarnata* were associated with significantly greater reductions in BDZ dosage at three months (CBT: *p* = 0.005, effect size: 0.032; *P. incarnata*: *p* < 0.001, effect size: 0.128). A significant interaction between CBT and *P. incarnata* was also observed (*p* = 0.037, effect size: 0.018), indicating a synergistic effect when both interventions were combined. Baseline sociodemographic characteristics, BDZ and antidepressant dosage and symptom severity did not differ significantly between groups. Patients taking 400–600 mg of *P. incarnata* dry extract showed a higher BDZ reduction compared to those taking 200 mg. **Conclusions**. These findings suggest that *P. incarnata* and CBT exert independent yet complementary effects in supporting BDZ tapering. Their combination appears to enhance dose reduction beyond either intervention alone, supporting a multimodal approach that addresses both neurobiological and psychological components of BDZ addiction. Prospective controlled studies are needed to confirm these results and to clarify their impact on long-term discontinuation outcomes.

## 1. Introduction

Benzodiazepines (BDZs) are among the most widely prescribed pharmacological treatment for the management of anxiety and insomnia, due to their rapid onset of action and their good short-term tolerability [1]. Their clinical properties are mediated primarily through positive allosteric modulation of the GABAA receptor, enhancing inhibitory neurotransmission and producing anxiolytic, sedative and muscle-relaxant effects [2]. Although highly effective in the acute management of anxiety and sleep disturbances, BDZs have long been associated with significant concerns when used chronically, including tolerance, dependence, cognitive impairment and an increased risk of falls and accidents, particularly in older patients [3,4,5,6,7]. The persistence of BDZ use in the general population has been attributed not only to their pharmacological profile but also to psychological and behavioural mechanisms, whereby patients often report a perceived inability to function without BDZs, reinforcing continued use that is no longer aligned with actual clinical needs [8,9,10,11,12,13]. Withdrawal symptoms, anxiety rebound and the subjective fear of symptom relapse frequently complicate discontinuation attempts, generating a clinical scenario in which tapering becomes difficult to implement and sustain [14,15,16,17]. These factors have prompted increasing interest in strategies that may support tapering programs and mitigate the neurobiological and psychological discomfort associated with BDZ reduction [18,19]. In recent years, attention has shifted toward complementary pharmacological agents capable of modulating anxiety and withdrawal-related hyperexcitability without introducing additional risks of dependence [20]. Among these, *Passiflora incarnata* L., herba (*P. incarnata*) has emerged as a potential therapeutic option due to its documented anxiolytic properties and its interaction with the GABAergic system [21,22,23,24].

Non-BDZ GABAergic agents, including pregabalin and gabapentin, have been investigated as potential options for the management of BDZ tapering and withdrawal, with evidence indicating their usefulness in certain patient populations despite variability in reported outcomes [25,26,27]. Several other pharmacological agents have also been evaluated as adjunctive strategies to facilitate BDZ discontinuation and mitigate withdrawal-related symptoms [28,29]. Unfavorable outcomes have been reported for β-blockers such as propranolol, progesterone, the 5-hydroxytryptamine-3 receptor antagonist ondansetron, and the tricyclic antidepressant dothiepin [30,31,32,33]. Buspirone has produced mixed results when used as an adjunct during tapering, whereas imipramine, carbamazepine, valproate and trazodone have demonstrated greater efficacy than placebo in supporting BDZ reduction [20,34,35,36,37]. In addition, research has explored the use of melatonin and herbal remedies with anxiolytic and hypnotic properties, including Valeriana officinalis, Melissa officinalis and Eschscholzia californica Cham., yielding inconsistent findings [24,38].

Evidence suggests that the benzoflavonoid fraction of *P. incarnata* may act as a modulator at both postsynaptic GABAA receptors and presynaptic GABAB receptors, thereby influencing inhibitory neurotransmission through distinct but converging pathways [21,23,39,40]. Such mechanisms may contribute to a reduction in somatic arousal and a dampening of withdrawal-related symptoms, making *P. incarnata* a plausible adjunctive agent during BDZ tapering [21,41].

In our previous real-world study, conducted on a cohort of 186 patients undergoing benzodiazepine reduction, we observed that add-on treatment with *P. incarnata* was associated with a significantly faster decrease in BDZ dosage over a three-month period compared with standard tapering protocols [12]. These findings were further reinforced by a subsequent long-term follow-up, which demonstrated that *P. incarnata* was well tolerated, easily discontinued once BDZs had been withdrawn, and not associated with rebound symptoms or psychological dependence, thereby supporting its role as a short-term facilitator of BDZ tapering rather than as a substitute with its own dependence liability [42].

However, the long-term analysis also revealed an important clinical observation: among all the demographic and clinical variables examined, the presence of comorbid personality disorders (PDs) was the only factor distinguishing patients who successfully discontinued BDZs from those who did not [42]. This result highlighted the possibility that psychological and behavioural factors, rather than symptom severity or pharmacological resistance, may constitute the primary barrier to successful tapering in a subpopulation of patients [10]. Since cognitive-behavioural therapy (CBT) is specifically designed to address maladaptive beliefs, emotional dysregulation and avoidance behaviours—factors known to maintain or exacerbate BDZ addiction—this observation strongly suggested the need to investigate whether engagement in CBT might influence tapering outcomes within the same cohort [43,44].

This clinical observation may be interpreted in light of convergent neurobiological mechanisms underlying both psychotherapeutic and adjunctive anxiolytic interventions. *P. incarnata* has been shown to exert anxiolytic effects primarily through modulation of the GABAergic system, including interactions with GABAA receptors and inhibition of GABA reuptake, thereby enhancing inhibitory control within stress-responsive neural circuits [23,45]. Notably, CBT has also been associated with functional and neuroplastic changes in cortico-limbic networks implicated in emotion regulation, fear extinction and behavioural inhibition, processes partly mediated by GABAergic neurotransmission [46,47]. Within this framework, CBT-related gains in emotional and behavioural regulation may compensate for deficits in inhibitory control typically addressed pharmacologically, thereby helping to explain why psychological and behavioral factors—rather than clinical severity—played a central role in determining BDZ tapering success in patients with comorbid PDs.

The present study therefore expands upon the original study by integrating CBT as an additional variable of interest. Through retrospective examination of clinical records, we identified patients who were concurrently undergoing CBT during the tapering period and assessed its contribution to BDZ reduction, both independently and in combination with *P. incarnata*. The aim of the present study was to assess the efficacy of combined CBT and *P. incarnata* dry extract as an add-on treatment for BDZ tapering, evaluating their individual and interactive effects.

## 2. Results

The whole sample consisted of 186 patients undergoing a protocol of BDZ downtitration, 93 with the addition of a dry extract of *P. incarnata* (mean age: 54.6 ± 16.4), and 93 without any add-on treatment (mean age: 51.7 ± 13.1).

Baseline sociodemographic and clinical characteristics of the sample, stratified by treatment condition and by psychotherapy status, are displayed in Table 1. The groups did not differ significantly with respect to age, sex, educational level, baseline BDZ and antidepressant dosage or baseline psychometric scores.

We performed subgroup analysis on patients undergoing CBT to compare CBT duration between patients undergoing standard BDZ tapering and *P. incarnata* add-on, finding no differences between the groups (CBT duration: months, mean ± SD: standard BDZ tapering: 12.29 ± 5.75; *P. incarnata* add-on: 13.59 ± 6.79; Mann–Whitney U test *p*: 0.346).

**Figure 1:**
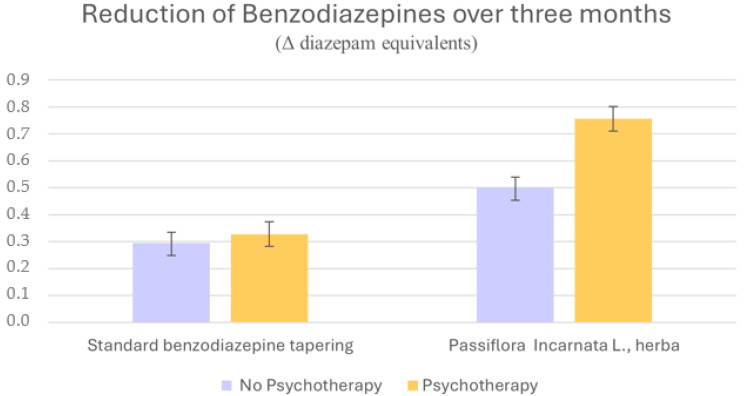
Comparison of benzodiazepine reduction (Δ mg diazepam equivalents) after three months between patients undergoing a standard BDZ tapering protocol and those treated with *P. incarnata*, with or without psychotherapy. Both psychotherapy and *P. incarnata* independently showed a significant effect on BDZ reduction, and they showed a synergistic effect when combined.

**Figure 2:**
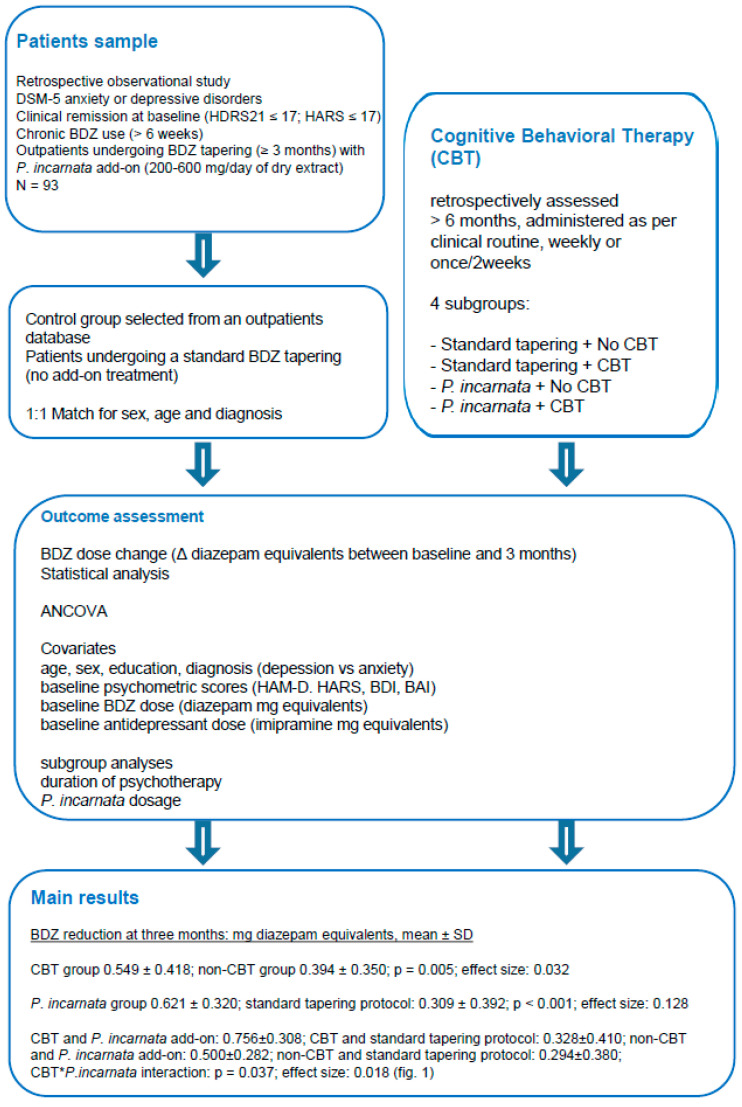
Flow-chart of study design and main results. DSM-5: Diagnostic and Statistical Manual of mental disorders, 5th edition; HDRS21: Hamilton Depression Rating Scale—21 items; HARS: Hamilton Anxiety Rating Scale; BDZ: benzodiazepine; *P. incarnata*: *Passiflora incarnata* L., herba; Δ mg diazepam equivalents: change in milligram diazepam equivalents; mg: milligram; BDI: Beck Depression Inventory; BAI: Beck Anxiety Inventory; SD: Standard Deviation.

The primary analysis focused on the reduction in BDZ dosage at three months, expressed as change of mg diazepam equivalents. The ANCOVA revealed a significant main effect of CBT on BDZ reduction (BDZ reduction at three months, mg diazepam equivalents, mean ± SD: CBT group 0.549 ± 0.418; non-CBT group 0.394 ± 0.350; *p* = 0.005; effect size: 0.032). A significant main effect of *P. incarnata* intake was also observed (BDZ reduction at three months, mg diazepam equivalents, mean ± SD: *P. incarnata* group 0.621 ± 0.320; standard tapering protocol: 0.309 ± 0.392; *p* < 0.001; effect size: 0.128). The analysis further indicated a significant CBT × *P. incarnata* interaction (BDZ reduction at three months, mg diazepam equivalents, mean ± SD: CBT and *P. incarnata* add-on: 0.756 ± 0.308; CBT and standard tapering protocol: 0.328 ± 0.410; non-CBT and *P. incarnata* add-on: 0.500 ± 0.282; non-CBT and standard tapering protocol: 0.294 ± 0.380; *p* = 0.037; effect size: 0.018). Covariates included sex, diagnosis, age, years of education, baseline scores of psychometric scales (HARS, HDRS21, BAI, and BDI), baseline BDZ dosage, baseline antidepressant dosage (see Section 4.4). Figure 1 displays the mean BDZ reduction across the four resulting subgroups defined by CBT status and *P. incarnata* use, illustrating the pattern of dosage change at three months. We summarized study design and main results in Figure 2.

To investigate the possible role of *P. incarnata* dosage on BDZ reduction, we performed an ANCOVA only on *P. incarnata* group, including sex, diagnosis, psychotherapy, *P. incarnata* dosage as fixed factors and age, education, baseline psychometric scores, BDZ and antidepressant baseline dosage as covariates. We did not find a significant effect of *P. incarnata* dosage on BDZ reduction (*p*: 0.090). However, we observed, accordingly with the previous work on the topic, a trend of higher BDZ reduction in patients taking 400 or 600 mg of *P. incarnata* dry extract compared with patients taking 200 mg. We thus considered a dichotomous variable (*P. incarnata* 200 mg vs. 400–600 mg). Performing the same ANCOVA analysis accordingly, we found a significant effect of *P. incarnata* dosage on BDZ reduction (*p*: 0.048; effect size: 0.031).

## 3. Discussion

BDZs remain among the most frequently prescribed psychotropic medications for the treatment of anxiety disorders, insomnia and mood disturbances, despite long-standing concerns regarding tolerance, dependence and the clinical complexity of long-term use [14]. Their continued widespread use reflects both their rapid anxiolytic and hypnotic effects and the difficulty many patients experience in managing distress without pharmacological support. However, prolonged BDZ exposure is associated with neuroadaptive changes within the GABAergic system, behavioral conditioning and psychological reliance, all of which contribute to the well-documented challenges of discontinuation [19]. In real-world clinical practice, BDZ tapering frequently proves more difficult than anticipated, even in patients who present with stable clinical features and are motivated to discontinue use.

As highlighted in our previous real-world study on BDZ tapering with and without *P. incarnata*, the difficulty of discontinuation rarely lies solely in the re-emergence of anxiety or insomnia [12]. Rather, it reflects a complex interaction between neurobiological adaptations induced by chronic BDZ exposure, psychological dependence on the medication as a primary coping strategy and behavioral reinforcement mechanisms that consolidate avoidance and reassurance-seeking patterns [11,13]. Over time, BDZs may become embedded in patients’ daily routines and identity as an indispensable aid to emotional regulation, thereby increasing anticipatory anxiety regarding dose reduction. These factors create a substantial barrier to tapering that is not adequately addressed by pharmacological dose reduction alone.

In this context, the identification of adjunctive interventions capable of facilitating BDZ reduction while minimizing withdrawal-related distress represents a clinically relevant goal. Our earlier findings suggested that *P. incarnata* may contribute meaningfully to this process, particularly during the initial phases of tapering. Patients receiving *P. incarnata* demonstrated a significantly faster reduction in BDZ dosage compared with those following standard tapering protocols, with the most pronounced effects observed at one month and persisting, though attenuated, at three months [12]. These findings are consistent with the hypothesis that *P. incarnata* plays a supportive role during the period of greatest neurophysiological instability and subjective discomfort.

The proposed mechanism underlying this effect relates to the pharmacodynamic properties of *P. incarnata*, particularly its benzoflavonoid components. Experimental data suggest that these compounds act as positive modulators of the postsynaptic GABAA receptor while simultaneously antagonizing presynaptic GABAB receptors [22,23,24,40]. This dual action may help stabilize GABAergic neurotransmission during BDZ withdrawal, reducing autonomic hyperarousal and somatic symptoms while potentially attenuating craving-like phenomena associated with reduced inhibitory tone. Preclinical studies support this interpretation, showing reduced withdrawal severity in diazepam-dependent animal models treated with *P. incarnata* extracts [39,41]. Moreover, clinical studies have demonstrated anxiolytic effects comparable to low-dose BDZs, without the cognitive impairment, psychomotor slowing or memory deficits commonly observed with BDZ use [21]. These characteristics distinguish *P. incarnata* from other pharmacological agents sometimes used in withdrawal management and support its suitability as a temporary adjunct rather than a long-term substitute.

Crucially, findings from the long-term follow-up conducted on the same cohort further reinforce this interpretation [42]. *P. incarnata* was readily discontinued once BDZ tapering had been completed, without evidence of rebound symptoms, withdrawal phenomena or psychological dependence. This observation is particularly relevant in the broader context of deprescribing, where the introduction of additional agents is often viewed with caution due to the risk of perpetuating pharmacological reliance. The absence of such effects suggests that *P. incarnata* may function as a facilitator of discontinuation rather than as a replacement dependency, enhancing patients’ tolerance to tapering during a critical transitional phase.

The present study extends these findings by examining the role of CBT, an intervention that was not systematically assessed in the original analysis. Psychotherapy represents a central component in the treatment of anxiety and depressive disorders and is particularly relevant to BDZ dependence, given its focus on modifying maladaptive cognitions, anticipatory anxiety and avoidance behaviors [44]. In our prior long-term follow-up, the presence of comorbid PDs emerged as the only variable differentiating patients who successfully discontinued BDZs from those who did not, despite similar levels of dose reduction. This finding suggested that psychological and behavioral factors, rather than residual symptom severity or pharmacological dependence alone, may represent the primary obstacles to complete discontinuation.

Against this background, evaluating the contribution of CBT within the same cohort represents a clinically meaningful extension of the original work. Through retrospective reconstruction of CBT exposure, we were able to assess its independent and interactive effects on BDZ reduction. Our findings show a significant main effect of CBT, as well as a significant interaction between CBT and *P. incarnata*. Interestingly, among patients undergoing a BDZ tapering regimen with *P. incarnata* add-on, patients taking 400–600 mg/day showed a higher BDZ reduction compared to patients taking 200 mg, consistent with our previous work.

These results suggest that pharmacological and psychotherapeutic interventions may target distinct components of BDZ dependence while mutually reinforcing their overall impact.

From a mechanistic perspective, *P. incarnata* may primarily address the physiological and neurochemical aspects of withdrawal, reducing somatic distress and facilitating early dose reductions. In contrast, CBT likely operates on the psychological and behavioral dimensions of dependence. By challenging interpretations of anxiety symptoms, reducing anticipatory fear associated with tapering and promoting alternative coping strategies, CBT may weaken the belief that BDZs are indispensable for emotional stability. Furthermore, CBT can help patients tolerate transient increases in distress without resorting to avoidance or medication escalation, thereby interrupting behavioral reinforcement cycles that sustain long-term use.

The significant interaction observed between CBT and *P. incarnata* suggests that these mechanisms may not simply be additive. Rather, the reduction of physiological withdrawal symptoms through pharmacological support may create a therapeutic window in which patients are more receptive to psychotherapeutic interventions. Conversely, CBT may enhance patients’ ability to make use of pharmacological support without developing new patterns of reliance. This reciprocal facilitation provides a plausible explanation for the observed acceleration of BDZ reduction when both interventions are present.

These findings also help contextualize the results of the long-term follow-up. Patients with comorbid PDs were able to reach low BDZ doses but showed lower rates of complete discontinuation, indicating that physiological dependence had largely been overcome, while psychological and behavioral barriers persisted. CBT, particularly when adapted to address emotion dysregulation, impulsivity and maladaptive interpersonal patterns, may therefore be particularly relevant for sustaining discontinuation beyond the tapering phase. The association between CBT exposure and greater dose reduction at three months aligns with this interpretation and supports a model in which combined pharmacological and psychological interventions improve both short-term tapering success and long-term outcomes.

### Limitations and Strengths

Some limitations must be acknowledged. First, the data collection was based on medical records, thus leading to a lack of possibly relevant clinical information. A placebo-controlled randomized design was not feasible in the present study because of its retrospective, observational, real-world nature. Treatment allocation reflected routine clinical practice and was guided by clinician judgment, patient preferences, and clinical characteristics, rather than by random assignment. Consequently, the inclusion of a placebo group was neither ethically nor practically possible, as all patients received standard-of-care interventions consistent with prevailing clinical guidelines at the time of treatment. CBT also was not randomly assigned, and thus differences in motivation, symptom perception or clinician decision-making cannot be fully excluded. Given the retrospective nature of the study, we could assess the compliance to the treatment only by asking patients themselves and caregivers.

On the other hand, our work has important strengths. The most relevant is its novelty, since only a few studies were conducted on the clinical effect of *P. incarnata* on large population samples. Nonetheless, the ecological validity of the study is strengthened by the naturalistic design, reflecting clinical practice where tapering strategies often integrate pharmacological and psychotherapeutic components in a non-standardized manner.

## 4. Materials and Methods

The present study extends the data of our previously published observational study, which included 93 outpatients undergoing BDZ tapering with add-on *P. incarnata* and 93 age- and sex-matched patients receiving a standard tapering protocol without adjunctive treatment. In this retrospective study only patients who underwent at least 3 months of tapering program were included. The patients were collected over an 18-month period (from July 2021 to December 2022) at Mood Disorder Unit at San Raffaele Hospital in Milan, Italy, representing a real-world target population. All participants met DSM-5 (Diagnostic and Statistical Manual of mental disorders, Fifth Edition) criteria for depressive or anxiety disorders, were in clinical remission at baseline, and were chronically treated with BDZs [48]. Sociodemographic characteristics, baseline psychometric assessments and BDZ and antidepressant dosages, expressed in diazepam and imipramine milligram equivalents, were collected at baseline and during the tapering process at one and three months. For the purposes of the present analysis, an additional variable was collected from clinical charts to determine whether patients were concurrently engaged in a structured CBT program during BDZ tapering. CBT status was recorded as a dichotomous measure based on documentation in the medical record. We selected only patients undergoing a CBT program since at least 6 months, with a reported good compliance and administered weekly or once every 2 weeks. We collected information about duration of CBT and used it as covariate in the analyses. The primary outcome of interest was the reduction in BDZ dosage at three months, calculated as the absolute change in diazepam milligram equivalents from baseline. To examine whether CBT contributed to benzodiazepine reduction independently of, or in combination with, *P. incarnata*, an analysis of covariance (ANCOVA) was performed. Sex, *P. incarnata* intake, diagnosis (anxiety vs. depression) and engagement in CBT were entered as fixed factors. Age, years of education, baseline scores on the Hamilton Anxiety Rating Scale (HARS), Hamilton Depression Rating Scale 21 items (HDRS21), Beck Anxiety Inventory (BAI) and Beck Depression Inventory (BDI), together with baseline BDZ and antidepressant dosage, were included as covariates. All statistical analyses were two-tailed, and statistical significance was set at *p* < 0.05.

### 4.1. Participants

A total of 93 euthymic patients undergoing a BDZ tapering regimen and receiving a dry extract of *P. incarnata*, for the management of anxiety symptoms were included in the study. *P. incarnata* was administered at a fixed daily dose ranging from 200 mg to 600 mg, as determined by clinical evaluation. Patients from this sample were individually matched to control subjects selected from a larger clinical database including our outpatient population undergoing a conventional BDZ tapering program based on a gradual weekly dose reduction, without any adjunctive treatment. Matching was performed manually on a 1:1 basis according to sex and diagnosis (exact matching) and age at enrolment, allowing a maximum difference of ±5 years. Inclusion criteria comprised age older than 17 years; a diagnosis of one of the included anxiety or depressive disorders according to DSM-5 [48]; clinical remission of depressive symptoms (HDRS21 ≤ 17) [49]; presence of mild anxiety symptoms (HARS ≤ 17) [50]; chronic BDZ use for more than six weeks; and ongoing treatment with a selective serotonin reuptake inhibitor (SSRI) or a serotonin–norepinephrine reuptake inhibitor (SNRI). Exclusion criteria included a diagnosis of psychotic disorders, current treatment with antipsychotics or mood stabilizers, and the presence of any substance use disorder, with the exception of BDZ misuse or abuse.

### 4.2. Passiflora incarnata L., Herba

This drug has been approved in Italy by the Italian Medicines Agency (AIFA, Agenzia Italiana del Farmaco, Roma, Italy) as a medicinal product since November 2020 (Tractana^®^, Baldacci Lab, Pisa, Italy). It is available in 200 mg tablets, with a maximum recommended daily dose of 1600 mg, and is routinely prescribed at our centre for the treatment of mild anxiety symptoms and sleep disturbances. In our outpatient setting, the most commonly adopted tapering protocol consists of a 25% dose reduction every two weeks, followed by a more gradual reduction of 12.5% every two weeks in the final phase of discontinuation. This schedule is nevertheless individualized according to each patient tolerability and the occurrence of anxiety rebound. None of the patients had been previously treated with Tractana^®^ prior to the start of the observational period.

### 4.3. Data Collection

At baseline, sociodemographic characteristics, current pharmacological treatments and clinical diagnoses were recorded. BDZ dosages at each assessment point were converted into diazepam milligram equivalents (hereafter referred to as mg-equivalents). For the purposes of the present study, information regarding the administration of CBT was retrospectively collected. Anxiety and depressive symptoms were assessed using both clinician-rated and self-report measures, including HARS, HDRS21, BAI, and BDI [49,50,51,52]. Anamnestic data were obtained from patients’ medical records at baseline (T0), while psychometric assessments and BDZ intake were evaluated at baseline, after one month (T1), and after three months (T2) from the initiation of BDZ dose reduction. All anamnestic and psychometric assessments were conducted by a trained psychiatrist. The study was approved by the Hospital Ethics Committee and conducted in accordance with the Declaration of Helsinki; all patient data were handled confidentially and anonymized.

### 4.4. Statistical Analyses

All statistical analyses were conducted using JASP software (version 0.16.4). Tables and figures were generated using either JASP or Microsoft Excel (version 15.59). All statistical tests were two-tailed, with the threshold for statistical significance set at *p* < 0.05. Continuous variables are presented as mean ± standard deviation (SD), whereas categorical variables are reported as frequencies and percentages. The Shapiro–Wilk test was used to assess the normality of continuous variables. Group comparisons for categorical variables were performed using chi-square tests. CBT exposure was coded as a dichotomous variable based on documentation in the medical records. All patients who met the inclusion criteria and were referred to our outpatient service during the observation period were included in the analyses. The primary outcome measure was the reduction in BDZ dosage at three months, defined as the absolute change in mg diazepam equivalents from baseline. To investigate whether CBT contributed to BDZ dose reduction independently or in interaction with *P. incarnata*, an analysis of covariance (ANCOVA) was performed. Sex, diagnosis, *P. incarnata* treatment and CBT engagement were entered as fixed factors, while age, years of education, baseline HARS, HDRS21, BAI and BDI scores, as well as baseline BDZ and antidepressant dosage, were included as covariates.

## 5. Conclusions

In conclusion, the present findings highlight the importance of combining biological and psychological interventions in the management of BDZ discontinuation. *P. incarnata* and CBT each exert independent effects but show a potentiated impact when used together. From a clinical perspective, the combined evidence across the two original studies and the present analysis suggests that *P. incarnata* and CBT may represent a useful multimodal strategy for supporting BDZ tapering in a real-world population. The pharmacological properties of *P. incarnata* appear to provide short-term relief from the physiological and neurobiological stressors of withdrawal, while CBT may address the cognitive, emotional and behavioural domains that sustain long-term use. Adopting such an integrated approach may be particularly valuable for patients with personality vulnerabilities, chronic BDZ intake or previous unsuccessful attempts at tapering. Future prospective studies with controlled designs are needed to clarify the temporal sequencing of these effects, determine whether integrated intervention models improve long-term abstinence rates, and further characterize the patient subgroups most likely to benefit from multimodal treatment strategies.

## Figures and Tables

**Table 1:** Sociodemographic and clinical data in patients undergoing a standard benzodiazepine tapering protocol or an add-on with *P. incarnata*, with or without CBT (Cognitive Behavioural Therapy). Abbreviations: HAM-A: Hamilton Anxiety Rating Scale; HAM-D: Hamilton Depression Rating Scale; BDI: Beck Depression Inventory; BAI: Beck Anxiety Inventory.

Standard Benzodiazepine Tapering			
	No CBT (N = 52)	CBT (N = 41)	*p* value
Age (mean ± SD)	51.06 ± 13.72	50.88 ± 13.59	0.517 ^a^
Sex (% of female)	69.23%	61.54%	0.631 ^c^
Education years (mean ± SD)	12.33 ± 3.44	12.98 ± 3.42	0.417 ^b^
HAM-A tot T0 (mean ± SD)	6.54 ± 4.58	6.10 ± 5.04	0.724 ^a^
HAM-D tot T0 (mean ± SD)	4.69 ± 3.5	4.59 ± 3.94	0.977 ^a^
BDI tot T0 (mean ± SD)	4.87 ± 3.71	4.83 ± 4.04	0.869 ^b^
BAI tot T0 (mean ± SD)	5.54 ± 4.47	5.66 ± 4.89	0.964 ^b^
Baseline BDZ dosage (diazepam mg equivalents; mean ± SD)	14.28 ± 8.05	16.04 ± 7.59	0.517 ^a^
Baseline antidepressant dosage (imipramine mg equivalents, mean ± SD)	111.79 ± 60.09	116.23 ± 56.97	0.554 ^b^
*P. incarnata* add-on			
	No CBT (N = 49)	CBT (N = 44)	*p* value
Age (mean ± SD)	56.27 ± 14.41	52.75 ± 18.3	0.977 ^a^
Sex (% of female)	68.25%	73.08%	0.653 ^c^
Education years (mean ± SD)	13.61 ± 3.45	13.23 ± 3.7	0.415 ^b^
HAM-A tot T0 (mean ± SD)	6.47 ± 4.45	6.39 ± 5.14	0.810 ^b^
HAM-D tot T0 (mean ± SD)	4.67 ± 3.44	4.71 ± 4.07	0.859 ^b^
BDI tot T0 (mean ± SD)	4.8 ± 3.4	5 ± 4.33	0.737 ^b^
BAI tot T0 (mean ± SD)	5.9 ± 4.27	5.59 ± 5.16	0.669 ^b^
Baseline BDZ dosage (diazepam mg equivalents; mean ± SD)	12.07 ± 10.08	15.14 ± 13.69	0.476 ^b^
Baseline antidepressant dosage (imipramine mg equivalents, mean ± SD)	109.35 ± 43.93	104.63 ± 55.90	0.515 ^b^

^a^ Student *t* test; ^b^ Mann–Whitney U test; ^c^ Chi-Squared test.

## Data Availability

The original contributions presented in this study are included in the article. Further inquiries can be directed to the corresponding author.

## Abbreviations

The following abbreviations are used in this manuscript:

*P. incarnata*	*Passiflora incarnata* L., herba
BDZ	Benzodiazepine
CBT	Cognitive-Behavioural Therapy
PD	Personality Disorder
HARS	Hamilton Anxiety Rating Scale
HDRS21	Hamilton Depression Rating Scale—21 items
BAI	Beck Anxiety Inventory
BDI	Beck Depression Inventory
DSM-5	Diagnostic and Statistical Manual of mental disorders—5th edition
SD	Standard Deviation
ANCOVA	ANalysis of COVAriance
